# Comparative analysis of choroidal thickness in third trimester pregnant women

**DOI:** 10.1186/s40942-018-0108-0

**Published:** 2018-01-29

**Authors:** Camila Zanella Benfica, Teresinha Zanella, Lucas Brandolt Farias, Maria Lúcia Rocha Oppermann, Luis Henrique Santos Canani, Daniel Lavinsky

**Affiliations:** 1 Universidade Federal do Rio Grande do Sul (UFRGS), Porto Alegre, Brazil; 2 Department of Ophthalmology, Hospital de Clínicas de Porto Alegre, Porto Alegre, Brazil; 3 Department of Gynecology and Obstetrics, Hospital de Clínicas de Porto Alegre, Porto Alegre, Brazil; 4 Department of Endocrinology, Hospital de Clínicas de Porto Alegre, Porto Alegre, Brazil

**Keywords:** Choroidal thickness, Enhanced depth imaging optical coherence tomography, Pregnancy, Choroid

## Abstract

**Background:**

The impact of pregnancy on the choroid is still under investigation. The aim of this study is to compare choroidal thickness measurements of healthy pregnant women in the third trimester and healthy non-pregnant women using spectral-domain optical coherence tomography (OCT).

**Methods:**

This cross-sectional study included 122 eyes of 61 women, divided into two groups: 27 healthy pregnant women in the third trimester and 34 age-matched healthy non-pregnant women. Choroidal thickness was measured using Enhanced Depth Imaging OCT at ten different locations: at the fovea and every 500 µm from the fovea up to 2500 µm temporally and up to 2000 µm nasally.

**Results:**

There were no significant differences in the ten measurements of choroidal thickness comparing both groups. Mean subfoveal choroidal thickness was 304.1 + 9.6 µm in the control group and 318.1 + 15.6 µm in the pregnant women group (p = 0.446). There was also no statistically significant association between gestational age and choroidal thickness measurements in the healthy pregnant women group.

**Conclusions:**

Our study showed no statistically difference in choroidal thickness between healthy non-pregnant women and healthy pregnant women in the third trimester.

## Background

Physiological changes during pregnancy are significant and their knowledge is essential to optimize outcomes. Volemia augmentation in pregnancy averages 40–45% above the nonpregnant blood volume after 32–34 weeks. Cardiac output is increased as early as the 5th week and reflects a reduced systemic vascular resistance and an increased heart rate [1–3].

Ocular changes during pregnancy like an increased central corneal thickness and curvature and decreased corneal sensitivity and intraocular pressure (IOP) were already described [4, 5]. Changes in ocular blood flow may also occur, as an increased pulsatile ocular blood flow [6].

The impact of pregnancy on the choroid, however, is still under investigation, with mixed results. A strong association of central serous chorioretinopathy and pregnancy is well documented [7, 8]. Choroidal dysfunction and ischemia are also a common ocular complication of preeclampsia [9].

The development of the enhanced depth imaging (EDI) technique of spectral-domain optical coherence tomography (SD-OCT) systems allowed analysis of choroidal morphologic features in normal and pathological eyes [10]. EDI-OCT promotes better documentation of the choroid and choroidal–scleral interface by decreasing signal strength posterior to the retinal pigment epithellium. Since it is a noninvasive diagnostic method, EDI-OCT would be ideal for the study of choroid changes during an uncomplicated pregnancy.

The aim of this study was to compare choroidal thickness measurements of healthy women, pregnant and non-pregnant, using EDI-OCT.

## Methods

This cross-sectional study included 122 eyes of 61 women, divided into two groups: 27 healthy pregnant women in the third trimester and 34 age-matched healthy non-pregnant women. The participants were recruited between March and September of 2016 at Hospital de Clinicas de Porto Alegre (HCPA), Brazil. All participants received in person full explanation about the study and provided written informed consent. This study was approved by HCPA research ethics committee and was conducted in accordance with the Declaration of Helsinki guidelines.

Participants underwent an interview with demographic and background history and complete ophthalmic examination. Subjects with any previous ocular surgery or ocular pathology including refractive disorders with spherical equivalent greater than ± 1.0 diopters were excluded. All pregnant women enrolled in the study were attending prenatal care and were having uneventful singleton pregnancy. Participants with history of smoking or diagnosed with any systemic disease, such as diabetes mellitus, hypertension, preeclampsia, renal, rheumatologic or cardiovascular diseases, were also excluded.

All OCT scans were performed in the morning (8:00 am to 12:00 pm) to avoid diurnal variations of choroidal thickness [11, 12]. The same experienced ophthalmologist (CB) performed all OCT scans, using Heidelberg Spectralis OCT (Heidelberg Engineering Co, Heidelberg, Germany). Choroid was imaged with a 6-line radial scan (30°, 9.2 mm) using EDI setting, with 100 images averaged per section. All scans were reviewed before their inclusion in the study; those with image artefacts or inaccurate choroidal limits were excluded. Only one single horizontal scan through the fovea was used for analysis.

**Fig. 1:**
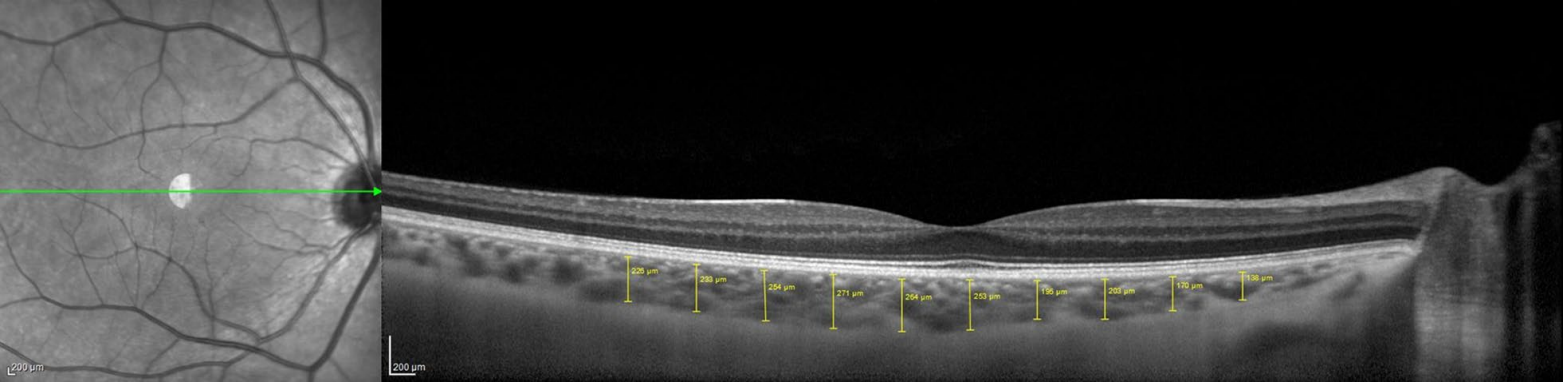
Measurements of choroidal thickness. Choroidal thickness measured at ten different locations: at the fovea and every 500 µm from the fovea up to 2500 µm temporally and up to 2000 µm nasally

Choroidal thickness was determined as the vertical distance from the outer surface of the line formed by the retinal pigment epithelium to the chorioscleral interface using the Spectralis OCT measurement software. The measurements were made by an experienced ophthalmologist (DL) masked to the participant group. Previous studies have already demonstrated the reproducibility of choroidal thickness measurements, even across different OCT systems [13–15]. Choroidal thickness was measured at ten different locations: at the fovea and every 500 µm from the fovea up to 2500 µm temporally and up to 2000 µm nasally (Fig. 1). We used the following abbreviations for the macular points: T5: choroidal thickness at 2500 µm temporally to the fovea; T4: choroidal thickness at 2000 µm temporally to the fovea; T3: choroidal thickness at 1500 µm temporally to the fovea; T2: choroidal thickness at 1000 µm temporally to the fovea; T1: choroidal thickness at 500 µm temporally to the fovea; SF: choroidal thickness at the fovea; N1: choroidal thickness at 500 µm nasally to the fovea; N2: choroidal thickness at 1000 µm nasally to the fovea; N3: choroidal thickness at 1500 µm nasally to the fovea; N4: choroidal thickness at 2000 µm nasally to the fovea.

### Statistical analysis

Statistical analyses were performed using SPSS V.15.0 (SPSS Science, Chicago, Illinois, USA). Quantitative variables from sample demographics were presented as mean ± SD. To compare variables between groups a t test was used for quantitative data and a Fisher’s exact test for qualitative data. Choroidal thickness measures were presented as mean ± SE. Differences in choroidal thickness were analysed using generalized estimating equations (GEE) with Bonferroni adjustment. Pearson’s correlation was used to analyze the relationship between choroidal thickness and gestational age. A p value ≤ 0.05 was considered statistically significant.

## Results

This study included 68 eyes of 34 healthy non-pregnant women (Control Group) and 54 eyes of 27 healthy pregnant women in the third trimester (Pregnant Group). Mean age of non-pregnant and pregnant women was 26.8 ± 5.0 and 28.1 ± 7.0 years, respectively (p = 0.439; t test). Racial distribution included 31 (91.2%) caucasians and 3 (8.8%) african-american in control group and 25 (92.6%) caucasians and 2 (7.4%) african-american in pregnant group (p = 1.000; Fisher’s exact test). The mean gestational age in the pregnant group was 33.3 ± 2.6 weeks.

The OCT scans were performed in all 54 eyes of the 27 healthy pregnant women and in all 68 eyes of the 34 healthy non-pregnant women. There were no significant differences in the ten measurements of choroidal thickness across the groups. (Table 1) Mean subfoveal choroidal thickness was 304.1 + 9.6 µm in the control group and 318.1 + 15.6 µm in the pregnant group (p = 0.446).

**Table 1:** Choroidal thickness measurements of healthy pregnant women in the third trimester and control group

	Control group	Pregnant group	p value
	Mean + SE (µm)	Mean + SE (µm)	
T5	277.4 + 9.6	278.5 + 13.9	0.949
T4	284.8 + 10.1	291.5 + 15.2	0.716
T3	292.1 + 9.8	300.9 + 14.3	0.611
T2	301.7 + 8.9	308.2 + 14.8	0.708
T1	299.1 + 8.6	311.2 + 14.8	0.478
SF	304.1 + 9.6	318.1 + 15.6	0.446
N1	285.1 + 9.6	291.3 + 14.9	0.730
N2	270.3 + 10.6	267.1 + 14.7	0.860
N3	245.6 + 10.9	239.4 + 13.9	0.725
N4	213.5 + 11.5	210.2 + 12.2	0.843

GEE with Bonferroni adjustment

T5: choroidal thickness at 2500 µm temporally to the fovea; T4: choroidal thickness at 2000 µm temporally to the fovea; T3: choroidal thickness at 1500 µm temporally to the fovea; T2: choroidal thickness at 1000 µm temporally to the fovea; T1: choroidal thickness at 500 µm temporally to the fovea; SF: choroidal thickness at the fovea; N1: choroidal thickness at 500 µm nasally to the fovea; N2: choroidal thickness at 1000 µm nasally to the fovea; N3: choroidal thickness at 1500 µm nasally to the fovea; N4: choroidal thickness at 2000 µm nasally to the fovea

We also analyzed if there was any correlation between the choroidal thickness measurements of both eyes and the gestational week in the third trimester of gestation using Pearson’s correlation. There was no statistically significant association between the gestational week and choroidal thickness measurements of both eyes in healthy pregnant women in the third trimester (Table 2).

**Table 2:** Correlation of gestational week and choroidal thickness measurements of both eyes in third trimester healthy pregnancies

	Pearson correlation coeficiente (r)	p value
T5		
OD	− 0.345	0.085
OS	− 0.202	0.322
T4		
OD	− 0.228	0.263
OS	− 0.167	0.414
T3		
OD	− 0.234	0.250
OS	− 0.093	0.650
T2		
OD	− 0.223	0.274
OS	− 0.073	0.723
T1		
OD	− 0.200	0.327
OS	− 0.141	0.492
SF		
OD	− 0.193	0.345
OS	− 0.213	0.295
N1		
OD	− 0.237	0.243
OS	− 0.216	0.289
N2		
OD	− 0.199	0.330
OS	− 0.180	0.378
N3		
OD	− 0.144	0.482
OS	− 0.149	0.468
N4		
OD	− 0.089	0.665
OS	− 0.143	0.487

T5: choroidal thickness at 2500 µm temporally to the fovea; T4: choroidal thickness at 2000 µm temporally to the fovea; T3: choroidal thickness at 1500 µm temporally to the fovea; T2: choroidal thickness at 1000 µm temporally to the fovea; T1: choroidal thickness at 500 µm temporally to the fovea; SF: choroidal thickness at the fovea; N1: choroidal thickness at 500 µm nasally to the fovea; N2: choroidal thickness at 1000 µm nasally to the fovea; N3: choroidal thickness at 1500 µm nasally to the fovea; N4: choroidal thickness at 2000 µm nasally to the fovea

## Discussion

The choroid is a complex vascular network which provides vascular supply for the retinal pigment epithelium and outer retina layers. It also provides thermal stability for the ocular tissues, removes ocular waste and acts in the uveoscleral aqueous drainage and regulation of intraocular pressure [4, 16]. Choroidal circulation is characterized by a high blood flow controlled by autonomic innervation, while retinal blood flow is mainly determined by autoregulatory mechanisms and local factors [17]. This vascular network is responsible for more than 85% of the blood flow in the eye and it can be influenced by hemodynamic factors such as blood flow and perfusion pressure [18].

Pregnancy itself promotes metabolic, hormonal and hemodynamic changes which could lead to changes in choroidal blood flow. During pregnancy there is an expansion of blood volume up to 45%, an increase in cardiac output and renin and angiotensin levels, and a decrease in colloid osmotic pressure, vascular resistance and arterial blood pressure [1, 3]. There are also some conditions such as central serous chorioretinopathy (CSC) which has an increased prevalence during pregnancy, especially in the third trimester [7, 19]. Previous studies have shown that patients with CSC have thickening of choroid when compared to controls [20, 21]. Choroidal vasodilation and vascular hyperpermeability can cause subsequent vascular leakage and increased hydrostatic pressure in the choroid. The high plasma cortisol concentration may also be a contributor for CSC development in pregnancy. All considered, it is essential to ask whether pregnancy itself can change choroidal structure and thickness.

Traditional imaging modalities such as indocyanine green angiography and Doppler ultrasonography were used in the past to assess choroidal function during pregnancy [9, 22]. The development of EDI-OCT, however, provided a fast, noninvasive and safe method to analyze choroidal thickness. Choroidal thickness can be influenced by major factors such as age, refractive error and axial length (AL), with increasing age, AL and decreasing refractive diopter being associated with a reduction of choroidal thickness [23]. Previous authors have measured choroidal thickness during pregnancy, with conflicting results [16, 24–32]. Different methodology may justify these different results. Table 3 summarizes results of different studies comparing choroidal thickness measurements of healthy pregnant and non-pregnant women using EDI-OCT.

**Table 3:** Summarized results of different studies comparing choroidal thickness measurements of healthy pregnant and non-pregnant women using EDI-OCT

References	Subjects	Gestational age at exam	Mean subfoveal choroidal thickness (SFCT)	Conclusion
Takahashi et al. [27]	30 pregnant women 30 non-pregnant women	Third trimester	275 ± 84 µm 273 ± 92 µm	No significant difference in choroidal thickness between groups (p = 0.925)
Sayin et al. [25]	46 pregnant women 40 non-pregnant women	Variable 28.0 ± 5.8 weeks (range: 17–37 weeks)	368.6 ± 67.6 µm 334.8 ± 59.9 µm	SFCT in normal pregnant women was significantly thicker than in non-pregnant healthy women (p = 0.038)
Kara et al. [24]	100 pregnant women 100 non-pregnant women	Variable 27.3 ± 6.6 weeks (range: 15–38 weeks)	371.1 ± 61.8 µm 337.2 ± 62.4 µm	SFCT in normal pregnant women was significantly thicker than in non-pregnant healthy women (p < 0.01)
Atas et al. [26]	25 pregnant women 26 non-pregnant women	Over 28 weeks	387.2 ± 60.76 µm 322.35 ± 63.89 µm	SFCT in normal pregnant women was significantly thicker than in non-pregnant healthy women (p < 0.001)
Goktas et al. [29]	30 pregnant women in the first trimester 30 pregnant women in the second trimester 30 pregnant women in the third trimester 30 non-pregnant women	First trimester Second trimester Third trimester	362 ± 81 µm 395 ± 80 µm 368 ± 70 µm 335 ± 86 µm	SFCT was significantly thicker in pregnant women in the second trimester (p = 0.007)
Ulusoy et al. [16]	29 pregnant women 36 non-pregnant women	Third trimester 3 months after delivery	387.97 ± 59.91 µm 332.40 ± 26.02 µm 320.86 ± 59.18 µm	SFCT significantly increases during pregnancy and returns to normal range 3 months after delivery
Kim et al. [21]	14 pregnant women 21 non-pregnant women	Third trimester	274.23 ± 29.30 µm 264.95 ± 21.03 µm	No significant difference in choroidal thickness between groups (p = 0.325)
Dadaci et al. [30]	27 pregnant women 25 non-pregnant women	First trimester Third trimester	OD: 349.22 ± 82.11 µm OE: 341.30 ± 85.22 µm OD: 333.56 ± 76.61 µm OE: 326.93 ± 75.84 µm OD: 318.88 ± 53.13 µm OE: 310.60 ± 51.09 µm	Choroidal thickness measurements in the third trimester were significantly decreased in both eyes compared to first trimester measurements
Rothwell et al. [31]	12 pregnant women 12 non-pregnant women	Third trimester	319.58 ± 6.11 µm 287.58 ± 43.44 µm	Choroidal thickness in normal pregnant women was significantly thicker than in non-pregnant healthy women (p = 0.034)
Acmaz et al. [32]	24 pregnant women 38 non-pregnant women	After 24 weeks	393.77 ± 61.83 µm 322.49 ± 65.58 µm	Choroidal thickness in normal pregnant women was significantly thicker than in non-pregnant healthy women (p < 0.001)

Kara et al. [24], Sayin et al. [25] and Atas et al. [26] conducted studies comparing choroidal thickness of healthy pregnant women in different gestational ages with healthy non-pregnant women. The authors concluded that subfoveal choroid was significantly thicker in pregnant women. However, other studies did not find this difference in choroid thickness. Takahashi et al. [27] and Kim et al. [28] demonstrated that choroidal thickness was not significantly different when comparing pregnant women in their third trimester and healthy non-pregnant women.

Other authors attempted to evaluate choroidal thickness considering gestational age. Goktas et al. [29] conducted a study with 90 healthy pregnant women, 30 at each pregnancy trimester, and 30 non-pregnant healthy women. Choroidal was significantly thicker in second trimester pregnant women in comparison with non-pregnant women. Dadaci et al. [30] compared choroidal thickness measurements of 54 eyes of 27 healthy pregnant women with 50 eyes of 25 non-pregnant women. The pregnant women underwent two OCT scans, one in the first trimester and the other in the third trimester. Choroidal thickness was significantly decreased at all measured points during the third trimester compared to the first trimester. The measurements of the control group were not statistically different.

Ulusoy et al. [16] conducted a prospective study to analyze choroidal thickness in third trimester pregnant women and 3 months after delivery. The subfoveal choroidal thickness was significantly reduced after delivery. A different control group of non-pregnant women was also analyzed and showed significantly thinner choroid measurements in comparison with pregnant women. Rothwell et al. [31] used a different technique to analyze choroid structure by constructing volume macular maps for the 9 subfields defined by the Early Treatment Diabetic Retinopathy Study. The measurements of thickness and volume in the central subfield were significantly greater in third trimester pregnant patients than in non-pregnant patients.

In this study, there were no significant differences in choroidal thickness among the two groups in ten macular points. We also found no significant correlation between choroid thickness and gestational age. These findings did not confirm our initial hypothesis that choroid could be thicker at pregnancy by an overall increase in choroidal blood flow and a decrease in intraocular pressure. However, the results of other studies about choroidal thickness during uncomplicated pregnancy are conflicting, and our findings are similar to those of Takahashi [27] and Kim [28].

Our study has some limitations, such as the small number of subjects. In addition, the cross-sectional design allow us to analyze choroid characteristics only in the third trimester of pregnancy, which could explain our lack of difference, since some authors described thicker choroids specifically at first or second trimesters. More consistent results could be achieved with a longitudinal study of choroidal thickness during the three trimesters of pregnancy and postpartum period with a large number of subjects.

In conclusion, our study reinforces absence of statistical difference in choroidal thickness between healthy third trimester pregnant women and healthy non-pregnant women. Further prospective studies with a larger number of subjects should be performed during different gestational ages and also after delivery.
